# A phase 2, proof of concept, randomised controlled trial of berberine ursodeoxycholate in patients with presumed non-alcoholic steatohepatitis and type 2 diabetes

**DOI:** 10.1038/s41467-021-25701-5

**Published:** 2021-09-17

**Authors:** Stephen A. Harrison, Nadege Gunn, Guy W. Neff, Anita Kohli, Liping Liu, Abbey Flyer, Lawrence Goldkind, Adrian M. Di Bisceglie

**Affiliations:** 1 Pinnacle Clinical Research, San Antonio, TX USA; 2 Pinnacle Clinical Research, Austin, TX USA; 3 Covenant Research, Sarasota, FL USA; 4 Arizona Liver Health, Chandler, AZ USA; 5 Hightide Therapeutics, Rockville, MD USA; 6 Pacific Northwest Statistical Consulting, Woodlinville, WA USA; 7 Uniformed Services University of Health Sciences, Bethesda, MA USA

**Keywords:** Diabetes, Non-alcoholic steatohepatitis

## Abstract

Non-alcoholic steatohepatitis is frequently associated with diabetes and may cause progressive liver disease. Current treatment options are limited. Here we report on a prospective, randomised, double-blind, placebo-controlled trial of two doses of HTD1801 (berberine ursodeoxycholate, an ionic salt of berberine and ursodeoxycholic acid), versus placebo that was conducted in 100 subjects with fatty liver disease and diabetes (NCT03656744). Treatment was for 18 weeks with a primary endpoint of reduction in liver fat content measured by magnetic resonance imaging proton density fat fraction. Key secondary endpoints included improvement in glycemic control, liver-associated enzymes and safety. The pre-specified primary endpoint was met. Thus, subjects receiving 1000 mg twice a day of berberine ursodeoxycholate had significantly greater reduction in liver fat content than in placebo recipients (mean absolute decrease −4.8% vs. −2.0% (*p* = 0.011). Compared to placebo, subjects receiving this dose also experienced significant improvement in glycemic control as well as reductions in liver-associated enzymes and significant weight loss. Diarrhea and abdominal discomfort were the most frequently reported adverse events. We conclude that berberine ursodeoxycholate has a broad spectrum of metabolic activity in patients with presumed NASH and diabetes. It is relatively well tolerated and merits further development as a treatment for NASH with diabetes.

## Abstract (web summary)

Berberine ursodeoxycholate has been studied for its serum lipid and lipoprotein lowering effects. Here the authors report an 18-week phase 2, randomised, double-blind, placebo-controlled clinical trial that tested the effect of berberine ursodeoxycholate in patients with fatty liver disease and diabetes, and showed that the group taking the higher dose of the drug had reduced liver fat content.

## Introduction

Non-alcoholic steatohepatitis (NASH) is a necroinflammatory disease of the liver which may lead to fibrosis and possible progression to cirrhosis. NASH is often associated with obesity, diabetes, hypertension, and hyperlipidemia^1^ and is often complicated by an increased risk of atherosclerotic cardiovascular events in addition to progressive liver disease. Weight loss may result in improvements in the degree of liver injury, including hepatic fibrosis, and has salutary metabolic effects^2^. Unfortunately, weight loss is very difficult for many individuals to attain and maintain, so there remains a need for drug therapy for NASH. Ideally, adjuvant therapy to lifestyle change would not only improve NASH histopathology but would also have a meaningful impact on the co-morbidities associated with NASH^1,2^.

There are currently no approved therapies for NASH, although a number of pharmacologic approaches are being explored. Some of the agents in advanced clinical trials include obeticholic acid, a bile acid that acts as a farnesoid X receptor (FXR) agonist, aramchol, a liver-targeted SCD-1 inhibitor, and resmetirom, a thyroid hormone β agonist^3^. HTD1801 (berberine ursodeoxycholate or BUDCA) is different in structure and function compared to these agents. It is an ionic salt of berberine and ursodeoxycholic acid, representing a molecular entity that offers the possibility of combination therapy for NASH and some of its comorbidities in a single treatment^4^. BUDCA is ingested in the form of a salt and it is thought that the salt promptly dissociates within the gastrointestinal tract and that BBR and UDCA are differentially absorbed^4,5^. BUDCA has been shown in animal models to improve fatty liver disease^5,6^ and its putative benefits in NAFLD are based upon known beneficial effects of each of its two constituent molecules. Thus, BBR has been shown in previous studies to be lipid-lowering and to improve glycemic control in diabetics^7–9^. UDCA has been shown to reduce liver injury associated with NAFLD in some studies but not in others^10–13^. Furthermore, it appears that when administered in the form of an ionic salt, the actions of these two-component molecules may be enhanced, presumably via their increased solubility and hence bioavailability^4,5^.

This proof of concept study focused on patients with type 2 diabetes for several reasons. Firstly, diabetics are known to be at greater risk for NASH than non-diabetics. In many clinical trials of therapies for NASH, the majority of subjects have diabetes and, given published experience with BBR showing improvement in glycemic control, it was felt that BUDCA was more likely to exert positive metabolic effects^14,15^. The aim of this study was to determine the effect of two different doses of BUDCA on liver fat content (LFC) in patients with diabetes and presumed NASH when administered for 18 weeks.

Here, we show that the higher dose of BUDCA tested in this phase 2 study (1000 mg twice a day) results in a significant reduction in liver fat content after 18 weeks of treatment. This improvement in liver fat content is associated with improvement in glycemic control, the apparent improvement in hepatic inflammation and injury with reductions in serum levels of liver-associated enzymes, significant weight loss, and modest reductions in serum lipid levels. These results indicate that BUDCA offers promise in the management of NASH and its related comorbidities and should be further tested.

## Results

### Baseline characteristics

**Fig. 1:**
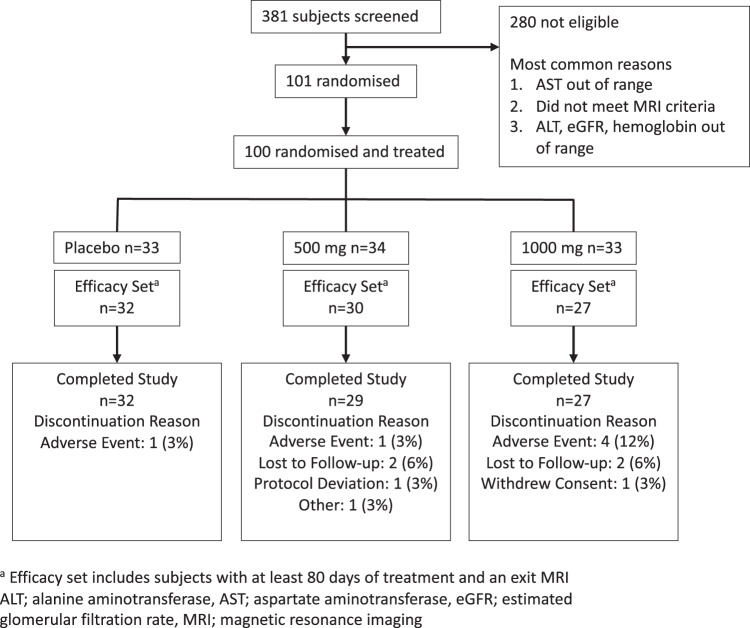
Clinical study flow diagram. Clinical study flow diagram showing the disposition of subjects with presumed NASH and diabetes screened and enrolled into a randomised controlled trial of berberine ursodeoxycholate (BUDCA).

There were 101 patients enrolled in this study. One did not meet entry criteria and was not dosed (Fig. 1). Of the remaining 100 subjects, their mean age was 56 years (range 26 to 75) and they included 72 females. Most of the subjects (91%) were white and 38% were of Hispanic ethnicity. Although all patients had a history of diabetes, the mean HbA1c level of those who enrolled was 7.1% (range 5.1 to 9.4%). Of note, 94 of 100 subjects were taking medication(s) for the treatment of their diabetes, often two or more agents at the same time. In addition, many were also taking lipid-lowering therapy as well (see Supplementary Table 1). Findings from multiparametric MRI showed that the mean liver fat content of the subjects was 19.4% at baseline (range 9.0 to 43.5%) and the mean corrected T1 was 940 (range 802 to 1412). The demographic and baseline characteristics of the subjects were evenly distributed across the three dosing groups (Table 1).

**Table 1:** Baseline characteristics.

	Treatment group		
	Placebo *N* = 33	500 mg BID *N* = 33	1000 mg BID *N* = 34
Age (years)			
* N*	33	33	34
Mean (SD)	58 (10.7)	58 (10.2)	53 (12.2)
Min, max	40, 75	26, 75	27, 72
Sex–*n* (%)			
Male	11 (33%)	7 (21%)	10 (29%)
Female	22 (67%)	26 (79%)	24 (71%)
Race–*n* (%)			
White	31 (94%)	29 (88%)	31 (91%)
Black	0	3 (9%)	2 (6%)
Other	2 (6%)	1 (3%)	1 (3%)
Ethnicity–*n* (%)			
Hispanic or Latino	13 (39%)	14 (42%)	11 (32%)
Not Hispanic or Latino	20 (61%)	19 (58%)	23 (68%)
Weight (kg)			
* N*	33	33	34
Mean (SD)	97.5 (22.57)	98.4 (23.05)	101.2 (20.26)
Min, max	70.8, 180	64.9, 154.1	71.7, 159.2
BMI (kg/m^2^)			
* N*	33	33	34
Mean (SD)	35.0 (6.18)	36.7 (6.88)	36.3 (6.28)
Min, max	25.4, 55.6	26.1, 55.9	25.4, 49.1
Liver fat content (%)*			
* N*	32	33	34
Mean (SD)	20.2 (6.23)	18.4 (6.24)	19.4 (6.96)
Min, max	11.1, 41.7	11.0, 43.48	9.0, 33.7
Corrected T1*			
* N*	32	32	32
Mean (SD)	940.8 (99.27)	948.8 (119.53)	932.4 (80.06)
Min, max	834.0, 1317.0	802.5, 1412.0	837.5, 1122.0
ALT (U/L)			
* N*	33	33	34
Mean (SD)	54 (26.7)	46 (27.6)	62 (31.8)
Median	47	39	55
Min, max	17, 115	3, 141	24, 143
AST (U/L)			
* N*	33	33	34
Mean (SD)	38 (17.3)	36 (15.9)	45 (29.7)
Median	32	31	37
Min, max	19, 88	15, 80	19, 147
GGT (U/L)			
* N*	33	33	34
Mean (SD)	70 (105.1)	64 (50.7)	68 (57.2)
Median	38	39	45
Min, max	19, 618	18, 223	19, 263
HbA1c (%)			
* N*	33	33	34
Mean (SD)	7.0 (1.05)	6.9 (0.85)	7.3 (1.16)
Median	6.9	6.9	7.5
Min, max	5.3, 9.4	5.5, 9.1	5.1, 9.3
LDL cholesterol (mg/dL)**			
* N*	33	30	29
Mean (SD)	99 (35.8)	86 (29.4)	107 (35.3)
Median	98	81	111
Min, max	37, 188	26, 150	31, 168
Triglycerides (mg/dL)**			
* N*	33	31	30
Mean (SD)	197 (83.3)	190 (204.6)	174 (77.1)
Min, max	76, 423	54, 1217	58, 430
Fasting insulin (IU/mL)			
* N*	33	33	34
Mean (SD)	45.7 (34.64)	30.8 (15.74)	32.9 (16.43)
Min, max	6.2, 155.4	9.5, 70.7	9.6, 82.4
Fasting plasma glucose (mg/dL)			
* N*	33	33	34
Mean (SD)	136 (44.1)	140 (39.9)	155 (46.3)
Min, max	94, 313	89, 288	90, 268

*Multiparametric MRI assessment.

**Modified efficacy set.

### Changes in liver fat content and liver-associated enzymes

Table 2 summarizes the changes seen with therapy, according to the treatment group. In general, the best treatment responses were seen with the higher dose of BUDCA (1000 mg BID). On average, absolute LFC decreased by 4.8% in this high dose group, compared to only 2.0% with placebo (*p* = 0.011). The relative decrease in LFC in this group was 24.1%, compared to 8.3% with placebo (*p* = 0.016). While there was an apparent dose-response with regard to the proportion achieving at least a 5% absolute decrease or a 30% relative decrease in LFC, these changes were not statistically significant. Other biochemical parameters associated with NASH and NAFLD also improved on therapy with this dose, as noted by significant decreases in serum alanine aminotransferase (ALT) and gamma-glutamyl transferase (GGT) levels.

**Table 2:** Responses to therapy.

	Treatment group		
	Placebo	500 mg BID	1000 mg BID
Absolute change in LFC (%)*			
* N*	32	30	27
Mean (SD)	−2.0 (4.88)	−2.9 (4.02)	−4.8 (4.35)
Min, max	−13.9, 6.3	−11.5, 5.9	−14.2, 2.9
LS mean (SE)	−1.8 (0.74)	−3.2 (0.77)	−4.7 (0.81)
Difference in LS means (SE) vs. placebo		−1.4 (1.06)	−2.9 (1.10)
* p*-value		0.199	0.011
Relative change in LFC (%)**			
* N*	32	30	27
Mean (SD)	−8.3 (24.48)	−15.1 (22.78)	−24.1 (21.70)
Min, max	−58.3, 46.6	−66.4, 35.3	−60.7, 22.1
LS mean (SE)	−8.2 (4.09)	−15.9 (4.27)	−23.3 (4.53)
* p*-value		0.196	0.016
Proportion achieving ≥5% absolute reduction in LFC**^a^	8/33 (24%)	10/31 (32%)	12/30 (40%)
*p*-value (Chi squared test)–post hoc		0.476	0.180
Proportion achieving ≥30% relative reduction in LFC**^a^	7/33 (21%)	6/31 (19%)	10/30 (33%)
*p*-value (Chi squared test)–post hoc		0.854	0.279
Mean change in HbA1c (%)**			
* N*	32	29	26
Mean (SD)	0.1 (0.82)	−0.3 (0.68)	−0.6 (0.96)
Min, max	−1.4, 2.7	−1.8, 1.3	−2.9, 1.6
LS mean (SE)	0.1 (0.14)	−0.4 (0.15)	−0.5 (0.16)
* p*-value		0.029	0.005
Mean change in ALT (U/L)**			
* N*	32	29	26
Mean (SD)	−3 (19.2)	−4 (17.9)	−19 (27.2)
Min, max	−50, 47	−33, 52	−89, 18
LS mean (SE)	−2 (3.5)	−5 (3.7)	−16 (3.8)
* p*-value		0.674	0.007
Mean change in GGT (U/L)**			
* N*	32	29	26
Mean (SD)	−2 (34.9)	−19 (26.4)	−30 (47.9)
Min, max	−120, 124	−104, 21	−213, 18
LS mean (SE)	−1 (4.6)	−20 (4.8)	−25 (5.0)
* p*-value		0.005	<0.001
Mean change in LDL-c (mg/dL)**			
* N*	29	27	25
Mean (SD)	0 (20.5)	5 (34.1)	−16 (26.5)
Min, max	−54, 48	−33, 147	−103, 31
LS mean (SE)	1 (5.3)	1 (5.4)	−12 (5.5)
* p*-value		0.955	0.072
Mean change in triglycerides (mg/dL)**			
* N*	32	29	26
Mean (SD)	18 (142.9)	−41 (136.3)	−24 (70.4)
Min, max	−242, 632	−710, 98	−161, 154
LS mean (SE)	19 (18.5)	−36 (19.4)	−24 (20.4)
* p*-value		0.041	0.120
Mean change in body weight (kg)***			
* N*	32	29	27
Mean (SD)	−1.1 (2.86)	−1.6 (3.02)	−3.5 (4.77)
Min, max	−9.1, 3.8	−7.2, 5.9	−18.9, 4.5
LS mean (SE)	−1.1 (0.64)	−1.6 (0.67)	−3.5 (0.70)
* p*-value		0.554	0.012

*Note*: Unless indicated otherwise statistical tests are two-sided p-values and LS Means are obtained from an ANCOVA model with treatment group as a fixed effect, and Baseline value of associated parameters as covariates. Tests are 1000 mg or 500 mg vs. placebo.

^a^A placebo subject had data available at Week 18/ET but did not have baseline data, so they were not included in the absolute and relative change from baseline analyses in the Efficacy Set.

*Primary endpoint results for efficacy set.

**Modified efficacy set.

***Safety set.

### Improvement in glycemic control, lipids, and weight loss

Notably, significant improvements were also seen in glycemic control with BUDCA therapy. Mean HbA1c levels decreased by 0.6% in the 1000 mg BID group and 0.3% in the 500 mg BID group compared to an increase of 0.1% in the placebo group. No significant changes were noted in levels of blood glucose, insulin, or HOMA-IR.

**Fig. 2:**
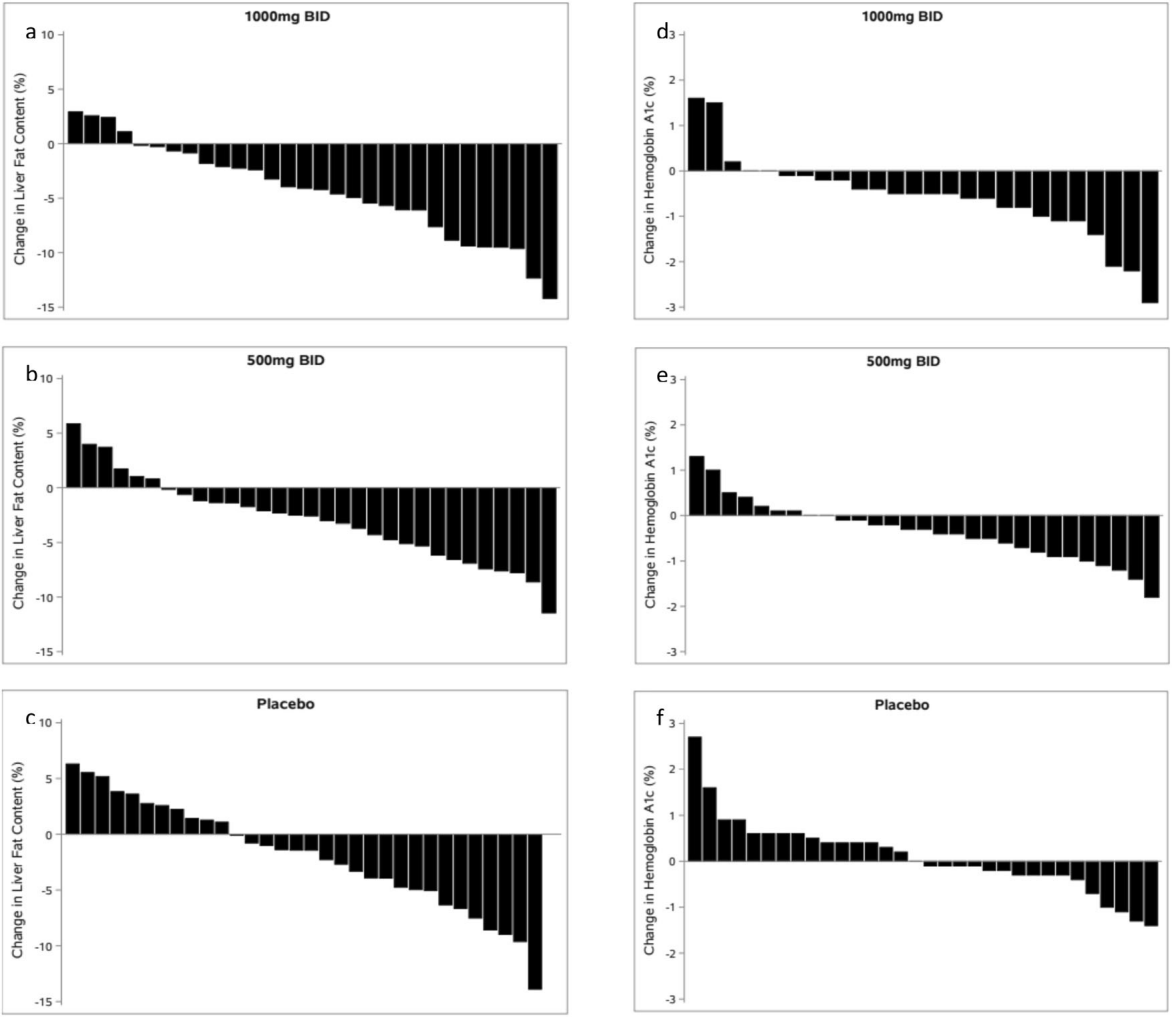
Waterfall plot of change in liver fat content and hemoglobin A1c in individual subjects. Liver fat content in panels **a** [*n* = 30], **b** [*n* = 31] and **c** [*n* = 33], and hemoglobin A1c (HbA1C) in panels **d** [*n* = 26], **e** [*n* = 29] and **f** [*n* = 32]), among subjects receiving berberine ursodeoxycholate (BUDCA) 1000 mg twice a day (BID), 500 mg BID or placebo, respectively.

Favorable decreases were also noted in plasma lipid and lipoprotein levels. Levels of LDL cholesterol decreased by an average of 16 mg/dL in the high dose group (*p* = 0.072) and triglyceride levels decreased by 41 mg/dL in the 500 mg BID dose group (*p* = 0.04), although not with the higher dose. There was no significant change in levels of HDL cholesterol (Supplementary Table 2). Finally, subjects receiving the higher dose lost an average of 3.5 kg in weight (*p* = 0.012). Figure 2 shows the decreases in liver fat content, HbA1c, and body weight in individual subjects.

Changes in serum levels of bile acids were noted (Supplementary Table 3) and were consistent with changes expected to occur with UDCA therapy. Supplementary Table 3 shows these changes from baseline in the highest dose group (1000 mg BID). While there was an increase in the total bile acids, levels of primary and secondary bile acids and their metabolites decreased, but a dramatic increase was noted in the levels of urso-bile acids and their metabolites. There were no significant changes in levels of fibroblast growth factor-19 (FGF19) or the serum fibrosis markers ELF and Pro-C3.

### Safety and tolerability

BUDCA therapy was generally well tolerated. Table 3 shows that the most frequent adverse event occurring during therapy was diarrhea while some subjects reported symptoms of gastrointestinal reflux or nausea. A similar number of subjects receiving either placebo or BUDCA reported having headaches. These adverse events were generally mild (grade 1 or 2, using CTCAE criteria). Only nine subjects had to discontinue BUDCA related to adverse events compared to only one on placebo, all but one of these being in the 1000 mg BID dosage group. Three serious adverse events occurred during the conduct of this study, one in a placebo recipient (bladder cancer) and two on BUDCA, neither of which were attributed to the study drug (one patient in the 500 mg BID group experienced a complication of low blood oxygen following elective shoulder surgery with an intercostal nerve block and the other, taking 1000 mg BID, had an acute myocardial infarction).

**Table 3:** Adverse events.

		Treatment group		
		Placebo *N* = 33	500 mg BID *N* = 33	1000 mg BID *N* = 34
Subjects with TEAEs related to study drug reported in two or more subjects in any treatment group	Diarrhea	0	4 (12%)	9 (26%)
	GERD	0	2 (6%)	0
	Nausea	0	1 (3%)	5 (15%)
	Headache	1 (3%)	2 (6%)	1 (3%)
Subjects with TEAEs requiring discontinuation of study drug	Diarrhea	0	0	2 (6%)
	GERD	0	1 (3%)	1 (3%)
	Abdominal distension	0	0	1 (3%)
	Melena	0	0	1 (3%)
	Acute myocardial infarction	0	0	1 (3%)
	Bladder cancer	1 (3%)	0	0
	Headache and facial rash	0	0	2 (6%)

## Discussion

This phase 2, randomised, placebo-controlled trial has shown that BUDCA is very effective in decreasing LFC in patients with presumed NASH and diabetes, as assessed by MRI-PDFF. Furthermore, BUDCA is also effective in improving glycemic control, measured by reductions in blood levels of HbA1c. In addition to these key benefits, improvements were seen in levels of liver-associated enzymes (ALT and GGT), as well as lipid levels (LDL-c and triglycerides).

The use of non-invasive tests such as MRI is now well established in early trials of agents to treat NASH^16,17^. Although liver biopsy was not a part of this study, the inclusion criteria allowed for an enrichment of the study population for NASH, the more severe form of fatty liver disease associated with inflammation and hepatic fibrosis. All subjects had to have at least 10% LFC by MRI-PDFF and in addition, they had to have corrected T1 values on MRI at a baseline of at least 830 msec. Furthermore, subjects were all diabetic and were required to have serum AST values of at least 20 U/L—both of these are risk factors for NASH among individuals known to have NAFLD. Reductions in liver fat content as assessed by MRI-PDFF have been shown to be predictive of histologic improvement in NASH^18^.

Each of the two parent compounds of BUDCA (berberine and ursodeoxycholic acid) is likely to have contributed to the beneficial effects seen in this patient population. Berberine is known to improve glycemic control and is commonly used as an over-the-counter remedy for diabetes and pre-diabetes^8,14^. Berberine has been shown to inhibit α-glucosidase in vitro and in animal models and this mechanism of action may have contributed further to the improvement seen in glycemic control^19,20^. The structural changes induced by berberine in the gut microbiome also have an effect on insulin resistance^15^. Based upon recently published findings, we hypothesize that berberine may act via activation of the intestinal farnesoid X receptor (FXR)^21^. This hypothesis is supported by the weight loss observed in this study and could be mediated by GLP-1 activation.

It is worthy of note that although their diabetes was relatively well-controlled, subjects enrolled in this study were heavily treated with other agents both before and during the study period, most commonly metformin, insulin, and GLP-1 agonists. Despite this intensive treatment, BUDCA provided significant further reductions in HbA1c levels. Berberine has previously been shown to reduce cholesterol levels (total and LDL-c) and this finding was confirmed in this study. Berberine acts by increasing the clearance of cholesterol by inducing the LDL receptor^8^. Although significant reductions were noted in triglyceride levels, the mechanism by which this occurs is not clear.

UDCA is not known to have any significant anti-diabetic activity at regular doses but does decrease serum cholesterol levels by decreasing cholesterol production^22^ and at high doses has been shown to lower HbA1C levels^9^. On the other hand, UDCA is well known to have substantial hepatoprotective effects^23,24^. For instance, in cholestatic diseases such as PBC, UDCA acts as a choleretic agent, increasing the flow of bile and also decreases the presence of toxic bile acids that accumulate in cholestasis^23^. Both berberine and UDCA appear to have anti-inflammatory effects, consistent with the reductions seen in serum levels of ALT and GGT in this study^24–27^ although these liver enzymes may have decreased simply because of the decrease in liver fat. Furthermore, both are able to modulate the gut microbiome in a favorable way^15,25^ and the microbiome has been found to play a role in the pathogenesis of NASH and obesity, related to diabetes^28,29^.

Although BUDCA is a single molecule, it is thought to dissociate into derivatives of its parent compounds in the gastrointestinal tract, each of which is differentially absorbed^4^. BUDCA is formed as a salt from equimolar amounts of berberine and UDCA. After the administration of BUDCA, increased levels of UDCA are noted in serum within about 2 h and persist for 7 h on average^4^. Levels of UDCA are about 1000 times higher in serum than berberine, on a molar basis, although animal studies have found an accumulation of berberine within the liver (data on file). The complex tertiary structure of the BUDCA salt appears to increase the bioavailability of berberine, both locally in the GI tract and systemically, perhaps accounting for the potent effect on glycemic control (data on file).

This study found that the use of BUDCA is also associated with significant weight loss (an average of 3.5 kg in the group dosed with 1000 mg BID). The mechanism of this weight loss is not clear. It is also uncertain how closely related the improvements in features of NASH are with weight loss. Several other agents currently being tested for NASH are also associated with weight loss (e.g., semaglutide)^30^.

BUDCA was relatively well tolerated. Gastrointestinal side effects including diarrhea and abdominal discomfort were the most frequently reported adverse event and necessitated discontinuation of study drug in about 12% of subjects. UDCA itself is associated with diarrhea and other GI complaints^31^. Berberine too can cause gastrointestinal symptoms, possibly via its inhibition of α-glucosidase, as occurs with some other anti-diabetes agents such as acarbose and miglitol^32^. Metformin too frequently causes GI upset^33^. The one serious adverse event occurring in the BUDCA high dose group was acute myocardial infarction thought not to be related to the study drug. It is well known that patients with NASH have a substantially increased risk of coronary artery disease and cardiovascular complications are the most frequent cause of death among patients with NASH^34^.

The use of BUDCA is associated not only with improvement in features of NASH (LFC, ALT, GGT) but also in other metabolic parameters including Hb1Ac and serum lipid levels. In contrast, some other agents being tested as a treatment for NASH may increase serum levels of cholesterol, often requiring concomitant therapy with an HMG CoA Reductase inhibitor (a “statin”) (e.g., obeticholic acid)^35^. Other agents (such as pioglitazone) may contribute to weight gain, whereas BUDCA is associated with weight loss^36^. Finally, it should be noted that BUDCA is orally administered whereas some other agents being developed require regular subcutaneous injection or even intravenous infusion^30,37^.

Subjects for this clinical trial were recruited at specialized liver centers in the United States. Findings from this study may perhaps not be extrapolated to the larger population of individuals with diabetes and fatty liver disease, both in the United States and beyond.

Two doses of BUDCA were tested in this study. While a dose-dependent effect was noted with some parameters, in general, the 500 mg BID dose was less effective than the 1000 mg BID dose. The adverse event profile was minimal and dose-dependent and the drug was generally well-tolerated.

In summary, BUDCA is a single molecule with a broad spectrum of metabolic activity in patients with fatty liver disease and presumed NASH. It is orally administered and relatively well tolerated. We conclude that data from this phase 2 randomised controlled trial support further development of BUDCA as a treatment for NASH in patients with diabetes and other features of the metabolic syndrome and we anticipate that the reduction in LFC will correlate with histopathologic improvement in future studies.

## Methods

### Study design and participants

Presumed NASH was defined based largely on magnetic resonance imaging derived proton density fat fraction (MRI-PDFF) of 10% or more^16^. Additional inclusion criteria further enriched the study population for having NASH by virtue of requiring corrected T1 (cT1) of more than 830 ms on MRI and elevated serum aspartate aminotransferase (AST) ≥20 units per liter^17^. Subjects had to be overweight or obese with a body mass index of 25 kg/m^2^ or more.

Study subjects were recruited at specialized liver centers around the United States between December 2018 and September 2019 and the last subject completed follow-up in March 2020. They underwent an initial screening evaluation, including measurement of liver fat content (LFC) by MRI-PDFF. Subjects were excluded if they had known liver disease other than fatty liver disease or a history of excessive alcohol consumption. Although liver biopsies were not done as a part of this study, subjects with clear evidence of cirrhosis were excluded, based upon a platelet count of less than 150,000/mm^3^, serum albumin levels less than 3.2 mg/dL, or current or previous history of clinical hepatic decompensation.

Subjects were permitted to continue their current treatment regimen for diabetes provided they had been on a stable dose regimen for at least 90 days. The treatment duration was for 18 weeks and study subjects were seen at intervals of 2 to 4 weeks throughout. LFC was measured at baseline and after 18 weeks of therapy. In addition, changes in liver chemistry tests, measures of glycemic control, and serum lipid levels were also assessed during and at the end of treatment.

The study was registered at clinicaltrials.gov (Identifier: NCT03656744). The protocol for this study was approved centrally by the WCG Institutional Review Board (WCG IRB, Payallup, Wa). All subjects gave written, informed consent for their participation. The results of this study are reported consistent with the CONSORT 2010 checklist^38^.

A pre-planned interim analysis was carried out as outlined in the protocol after 51 subjects had completed the assessment of the primary efficacy endpoint to assess sample size assumptions and futility through conditional power; this interim analysis did not include an option to stop early for efficacy and hence no alpha was spent. This interim analysis was conducted by an independent, unblinded statistician. The futility boundary was not crossed and so the study continued. The lower than anticipated standard deviation offset the lower than anticipated treatment effect. Following the rules for the primary efficacy endpoint, the sample size was not increased, and the study continued based on the original sample size. The results were not shared with the sponsor or investigators. The achieved sample size in the efficacy population (*n* = 89) was lower than the planned sample size (*n* = 105). This 15% decrease in sample size resulted in standard errors that were about 9% larger than they would have been with the full sample size. It is also of note that the observed treatment effect for 1000 mg (2.8566) was smaller than planned (5%). However, the observed standard deviations were smaller than planned for, allowing statistical significance to be reached.

Randomisation: Patients with diabetes and presumed NASH were randomised, using permuted blocks without additional stratification, into one of three treatment groups, in a 1:1:1 ratio as follows: (1) BUDCA 500 mg BID (2) 1000 mg BID, and (3) matching placebo. Through the use of a blinded randomisation number, subjects and investigators were isolated from the knowledge of treatment assignment. The randomisation allocation sequence was created by PharPoint Research (Wilmington, NC) within SAS (v9.4) using proc plan. The design was simple randomisation with a block size of 3, with 50 blocks generated for a total of 150 numbers. The numbering sequence, and similarly generated scrambled kit list, were then implemented by a central interactive web response system (IWRS) managed by Medidata Solutions (New York, NY). All subjects and study personnel, except for the unblinded statistician, the IWRS vendor, and the study drug packaging supplier, remained blinded to the treatment administered throughout the study. All study drugs were provided in matching white tablets, four tablets to a pouch, two pouches to be administered each day.

Statistical methods: Assuming a standard deviation of 6.3% for changes from Baseline in LFC, 35 subjects in each treatment group provided 90% power to show a difference of 5 percentage points between any 2 treatment groups at the 5% level of significance. To allow for a dropout rate of 10%, up to 39 subjects could have been randomised to each of the 3 treatment groups. The achieved sample size was 33 to 34 subjects per arm.

Descriptive Statistics were used to summarize continuous data, and frequencies and percentages were used to summarize categorical data. The Safety Set consisted of all subjects who received at least one dose of study treatment. The Modified Efficacy Set is a subset of the Safety Set consisting of subjects that had at least one post-dose MRI-PDFF assessment. The Efficacy Set is a subset of the Modified Efficacy Set consisting of subjects that completed at least 80 days of study drug dosing and had a week 18 or Early Termination (ET) visit MRI-PDFF assessment. The primary endpoint was an absolute change from Baseline to Week 18 (or ET) in Liver Fat Content. Primary Endpoint summary measures were provided in both the Efficacy and Modified Efficacy sets, with the Efficacy Set prespecified as the primary analysis set. Key secondary endpoints included improvement in glycemic control, liver-associated enzymes, and safety and were assessed using the Modified Efficacy Set. Comparisons between active treatment groups relative to placebo were tested using Analysis of Covariance (ANCOVA) with treatment group as a fixed effect and baseline value of the parameters of interest as covariates. ANCOVA-based LS Means and raw means have been reported. For statistical analysis of laboratory parameters (i.e., *p*-values), multiple imputations were used to take missing data into account. A two-sided alpha level of 0.05 without adjustment for multiple tests was used to indicate statistical significance. Analyses were performed using SAS System version 9.4.

### Reporting summary

Further information on research design is available in the Nature Research Reporting Summary linked to this article.

## Supplementary information

Supplementary Information

Reporting Summary

## Data Availability

The datasets generated and/or analyzed in this study are considered commercially sensitive and, therefore, are not publicly available. Requests for data supporting findings in the manuscript should be made to the corresponding author and will be reviewed individually. Data might be shared in the form of aggregate data summaries and via a data transfer agreement. Individual participant-level raw data containing confidential or identifiable patient information are subject to patient privacy and cannot be shared. Source data are provided with this paper.

## Source data

Source data

## Code availability

The treatment code cannot be made available due to proprietary reasons.
