# Supplementary material for: A phase 2, proof of concept, randomised controlled trial of berberine ursodeoxycholate in patients with presumed non-alcoholic steatohepatitis and type 2 diabetes
Source: Nat Commun. 2021 Sep 17;12:5503. doi: 10.1038/s41467-021-25701-5 (PMC8448729; doi:10.1038/s41467-021-25701-5)
Supplement: Supplementary file 1 — Supplementary Information [file 41467_2021_25701_MOESM1_ESM.pdf]

A Phase 2, Proof of Concept, Randomised Controlled Trial of Berberine Ursodeoxycholate in Patients with Presumed Non-Alcoholic Steatohepatitis and Type 2 Diabetes

Stephen A. Harrison[1], Nadege Gunn[2], Guy W. Neff[3], Anita Kohli[4], Liping Liu[5], Abbey Flyer[6], Lawrence Goldkind[7], Adrian M. Di Bisceglie[5]

[1]Pinnacle Clinical Research, San Antonio, Tx, [2]Pinnacle Clinical Research, Austin, TX, [3]Covenant Research, Sarasota, FL, [4]Arizona Liver Health, Chandler, AZ, [5]Hightide Therapeutics Inc, [6]Pacific Northwest Statistical Consulting, Woodlinville, WA, [7]Uniformed Services University of Health Sciences

Supplementary Table 1: Concomitant medications of interest, at baseline

		Treatment Group		
		Placebo<br>N=33	500mg BID<br>N=33	1000mg BID<br>N=34
For diabetes	Metformin	28 (85%)	24 (73%)	27 (79%)
	GLP-1 agonists	12 (36%)	6 (18%)	7 (21%)
	Sulfonylureas	8 (24%)	5 (15%)	6 (18%)
	Insulin/s	8 (24%)	1 (3%)	7 (21%)
	SGLT-2 inhibitors	3 (10%)	5 (15%)	3 (10%)
	DPP-4 inhibitors	3 (10%)	1 (3%)	5 (15%)
	Fixed dose combinations	4 (12%)	2 (6%)	1 (3%)
	Repaglinide	1 (3%)	0	0
	Pioglitazone	0	1 (3%)	0
For hyperlipidemia	HMG CoA Reductase Inhibitors	15 (45%)	23 (70%)	12 (35%)
	Fibrates	2 (6%)	4 (12%)	4 (12%)
	Fish Oil and Omega-3 Fatty Acids	9 (27%)	2 (6%)	5 (15%)
	Colesevalam	0	0	1 (3%)
	PCSK9 Inhibitors	1 (3%)	0	0
Other	Vitamin E	2 (6%)	0	0

Supplementary Table 2: Responses to therapy for additional endpoints* in the Modified Efficacy Set

	Treatment Group		
	Placebo	500mg BID	1000mg BID
Relative change in ALT (%)			
N	32	29	26
Mean (SD)	-6 (30.5)	-6 (36.0)	-21 (35.2)
Min, Max	-52, 69	-60, 89	-83, 45
Relative change in GGT (%)			
N	32	29	26
Mean (SD)	5 (39.7)	-23 (25.8)	-29 (27.1)
Min, Max	-46, 175	-62, 64	-81, 24
AST (U/L)			
N	32	29	27
Mean (SD)	-3 (11.6)	0 (13.2)	-12 (26.0)
Min, Max	-36, 19	-25, 35	-87, 16
HDL Cholesterol (mg/dL)			
N	32	29	27
Mean (SD)	-0.2 (7.47)	-0.3 (8.13)	1.2 (5.70)
Min, Max	--17, 23	-18, 25	-10, 15

*The additional endpoints listed in this table were not pre-specified in the Statistical Analysis Plan and hence no p Values were calculated

Supplemental Table 3: Change in Bile Acids from Baseline to Week 18 in 1000mg BID treatment group

	Baseline (ng/mL)	Absolute Change from Baseline (ng/mL)	Percent Change from Baseline
Total bile acids			
Mean (SD)	1864 (1750)	1625 (2332.2)	256 (455.8)
Min, Max	307, 6717	-3075, 6179	-46, 1437
1000mg vs Placebo ANCOVA p-value		0.016	
Primary bile acids and metabolites			
Mean (SD)	969 (900)	-171 (883.1)	114 (371.3)
Min, Max	132-3361	-1495, 1484	-71, 1118
1000mg vs Placebo ANCOVA p-value		0.882	
Secondary bile acids and metabolites			
Mean (SD)	445 (265)	-28 (300.6)	-13 (83.6)
Min, Max	139-1020	-486, 667	-100, 167
1000mg vs Placebo ANCOVA p-value		0.459	
Urso- bile acids			
Mean (SD)	449 (1350)	1824 (2122.6)	4741 (7699.7)
Min, Max	8-5132	-2787, 4964	-80, 27623
1000mg vs Placebo ANCOVA p-value		<0.001	

Note: Two-sided p-values are obtained from an ANCOVA model with treatment group as a fixed effect, and Baseline value of the given laboratory assessment as a covariate.
Primary bile acids include chenodeoxycholic acid, cholic acid and their taurine and glycine conjugates.
Secondary bile acids include deoxycholic acid, lithocholic acid and their taurine and glycine conjugates.
Urso- bile acids include ursodeoxycholic acid and its taurine and glycine conjugates.
