# Supplementary material for: A phase 2, proof of concept, randomised controlled trial of berberine ursodeoxycholate in patients with presumed non-alcoholic steatohepatitis and type 2 diabetes
Source: Nat Commun. 2021 Sep 17;12:5503. doi: 10.1038/s41467-021-25701-5 (PMC8448729; doi:10.1038/s41467-021-25701-5)
Supplement: Supplementary file 2 — Reporting Summary [file 41467_2021_25701_MOESM2_ESM.pdf]

nature research

NCOMMS-21-07631B

Corresponding author(s): Adrian Di Bisceglie

Last updated by author(s): Jul 17, 2021

Reporting Summary

Nature Research wishes to improve the reproducibility of the work that we publish. This form provides structure for consistency and transparency in reporting. For further information on Nature Research policies, see our Editorial Policies and the Editorial Policy Checklist.

Statistics

For all statistical analyses, confirm that the following items are present in the figure legend, table legend, main text, or Methods section.

n/a	Confirmed	
☐	☒	The exact sample size (n) for each experimental group/condition, given as a discrete number and unit of measurement
☐	☐	A statement on whether measurements were taken from distinct samples or whether the same sample was measured repeatedly
☐	☒	The statistical test(s) used AND whether they are one- or two-sided<br>*Only common tests should be described solely by name; describe more complex techniques in the Methods section.*
☐	☒	A description of all covariates tested
☐	☒	A description of any assumptions or corrections, such as tests of normality and adjustment for multiple comparisons
☐	☒	A full description of the statistical parameters including central tendency (e.g. means) or other basic estimates (e.g. regression coefficient) AND variation (e.g. standard deviation) or associated estimates of uncertainty (e.g. confidence intervals)
☐	☒	For null hypothesis testing, the test statistic (e.g. F, t, r) with confidence intervals, effect sizes, degrees of freedom and P value noted<br>*Give P values as exact values whenever suitable.*
☒	☐	For Bayesian analysis, information on the choice of priors and Markov chain Monte Carlo settings
☒	☐	For hierarchical and complex designs, identification of the appropriate level for tests and full reporting of outcomes
☒	☐	Estimates of effect sizes (e.g. Cohen's d, Pearson's r), indicating how they were calculated

Our web collection on statistics for biologists contains articles on many of the points above.

Software and code

Policy information about availability of computer code

Data collection	Medidata IWRS for Randomization and electronic case report forms (eCRF)<br>MedDRA version 20.1<br>WHO Drug Dictionary Enhanced September 2017 version
Data analysis	CTCAE version 4.0 for toxicity grading, and the following procedures were used in SAS Version 9.4: proc tabulate, proc freq, proc means, proc glm.

For manuscripts utilizing custom algorithms or software that are central to the research but not yet described in published literature, software must be made available to editors and reviewers. We strongly encourage code deposition in a community repository (e.g. GitHub). See the Nature Research guidelines for submitting code & software for further information.

Data

Policy information about availability of data

All manuscripts must include a data availability statement. This statement should provide the following information, where applicable:

- Accession codes, unique identifiers, or web links for publicly available datasets
- A list of figures that have associated raw data
- A description of any restrictions on data availability

Provide your data availability statement here.

April 2020

Field-specific reporting

Please select the one below that is the best fit for your research. If you are not sure, read the appropriate sections before making your selection.

☒ Life sciences ☐ Behavioural & social sciences ☐ Ecological, evolutionary & environmental sciences

For a reference copy of the document with all sections, see nature.com/documents/nr-reporting-summary-flat.pdf

Life sciences study design

All studies must disclose on these points even when the disclosure is negative.

Sample size	Assuming a standard deviation of 6.3% for changes from Baseline in LFC, 35 subjects in each treatment group provided 90% power to show a difference of 5 percentage points between any 2 treatment groups at the 5% level of significance. To allow for a dropout rate of 10%, 39 subjects were randomized to each of the 3 treatment groups.
Data exclusions	Subjects were excluded if they had known liver disease other than fatty liver disease or a history of excessive alcohol consumption. Although liver biopsies were not done as a part of this study, subjects with clear evidence of cirrhosis were excluded, based upon a platelet count of less than 150,000/mm3, serum albumin levels less than 3.2 mg/dL or a current or previous history of clinical hepatic decompensation.
Replication	This is a clinical trial, not possible to replicate. This is the first phase 2 trial with this investigational compound
Randomization	Subjects were randomized in a 1:1:1 ratio to each of the 3 treatment groups.
Blinding	This was a double blind study; all subjects, study personnel, and sponsor staff were blinded to the treatment administered through the study.

Reporting for specific materials, systems and methods

We require information from authors about some types of materials, experimental systems and methods used in many studies. Here, indicate whether each material, system or method listed is relevant to your study. If you are not sure if a list item applies to your research, read the appropriate section before selecting a response.

Materials & experimental systems

n/a	Involved in the study	
☒	☐	Antibodies
☒	☐	Eukaryotic cell lines
☒	☐	Palaeontology and archaeology
☒	☐	Animals and other organisms
☐	☒	Human research participants
☐	☒	Clinical data
☒	☐	Dual use research of concern

Methods

n/a	Involved in the study	
☒	☐	ChIP-seq
☒	☐	Flow cytometry
☒	☐	MRI-based neuroimaging

Human research participants

Policy information about studies involving human research participants

Population characteristics	Adults 18 to 75 years old, with clinical diagnosis of Nonalcoholic Steatohepatitis (NASH) as assessed by MRI, clinically documented diagnosis of Type 2 Diabetes Mellitus (T2DM), and BMI >25 kg/m2.
Recruitment	Subjects were recruited at specialized liver centers around the United States, therefore these data may not be applicable to the general population of persons with diabetes and fatty liver disease or to similar patients in other cpountries or regions of the world
Ethics oversight	Western Investigational Review Board (WIRB)

Note that full information on the approval of the study protocol must also be provided in the manuscript.

Clinical data

Policy information about clinical studies

All manuscripts should comply with the ICMJEguidelines for publication of clinical research and a completedCONSORT checklist must be included with all submissions.

Clinical trial registration	clinicaltrials.gov Identifier: NCT03656744
Study protocol	Study protocol is not publicly available for proprietary reasons.

Data collection	Study subjects were recruited at specialized liver centers around the United States between January and October 2019. Data from these sites was entered into a common electronic data capture system (eDC).
Outcomes	The primary endpoint for this study was the absolute change in LFC as measured by MRI-PDFF from Baseline to Week 18. The primary analysis for the difference in absolute change in LFC was an Analysis of Covariance (ANCOVA) including treatment as a fixed effect, and baseline LFC and ALT as covariates. Comparisons of each active treatment group to placebo within the Efficacy Set was tested at the 5% level of significance without multiplicity adjustment. LS mean differences between treatment groups were also produced with associated standard errors.<br>Secondary Endpoints included change from baseline and percent change from baseline to Week 18 within the Modified Efficacy Set for fasting glucose, HbA1c Proportion of subjects who achieve ≥ 30% relative reduction in LFC, LFC, Proportion of subjects who achieve ≥ 5% absolute reduction in LFC, (LDL-c), serum triglycerides, AST, ALT, GGT, total bile acid, total primary bile acids (and metabolites), total secondary bile acids (and metabolites), and total ursodeoxycholic acid (and metabolites). For relative change in LFC, an ANCOVA model including the effects of treatment group, baseline LFC and ALT was produced. For laboratory assessments, an ANCOVA model that included effects of treatment group and corresponding baseline lab value was produced, comparing each active treatment group to placebo.<br>Additional details are defined in the Study Protocol and Statistical Analysis Plan.
